# Modern Medical Training*Address delivered at the opening of the Medical Department of the University of Texas, November 15, 1900.

**Published:** 1900-12

**Authors:** H. P. Cooke

**Affiliations:** Galveston, Texas; Dean Medical Department, University of Texas


### TEXAS MEDICAL JOURNAL.

ESTABLISHED JULY, 1885.

PUBLISHED MONTHLY.—SUBSCRIPTION $1.00 A YEAR.

VOL. XVI. AUSTIN, DECEMBER, 1900. No. 6.

### Original Contributions.

For Texas Medical Journal.

### Modern Medical Training.*

BY H. P. COOKE, M. D., GALVESTON, TEXAS.
Dean Medical Department, University of Texas.

At the close of the period set aside for preparing the student for his future life work, and at the beginning of his entry into the field in which his learning is to be tested in actual practice, it is the custom to introduce him to the world through the medium of commencement exercises; and appropriately so, for he comes from the seclusion of hard study and training to commence his work out in the open, in the inevitable competition with his fellowmen for success in life. This is the public function, and is of equal interest to the novice and to those already ahead of him in the field.

There is another point in the student's career which, while public, is in a measure a personal, a family, affair with him and the teachers, who are largely to be responsible for his fitness when he is presented to the world at his commencement. I allude to the opening exercises observed on the day that both teacher and pupil begin work—the one to inspire, the other to learn and acquire.

A simple ceremony this usually becomes, notable for the directness of contact from speaker to audience; notable, too, for the

*Address delivered at the opening of the Medical Department of the University of Texas, November 15, 1900.

sympathy which enfolds everyone in the community of interests. Is it to be wondered that we keep this day of counseling to ourselves, and hedge ourselves somewhat apart from the outside world, which now seems so far from those most concerned—the new students? The introduction this time is to an individual fellow companion of the road before us; and not, as later, to the multitude from whom are to be selected the closer intimacies and associations of the larger life.

On this occasion it has befallen me to assume the pleasant task of speaking directly from your instructors to you. And, indeed, I am nothing loath to have a word or two with you, and to share some of the thoughts—largely in regard to yourselves—which have intruded themselves in the course of my connection with this school and with your predecessors. Your chosen profession has become so vast in its dimensions, so intricate in its composition, and so exacting in its demands that a close scrutiny has become necessary of many possible influences tending to make or mar the men who take it up. Previous preparation, personal fitness, habits of study, individual qualifications and a host of other things claim the attention in estimating your value as material for future doctors. Coming in contact with you, as I do, at the very threshold of your residence here, and then losing you to find you again almost at the open door of your departure, it has been most interesting to me to ascertain what each of you has done with the opportunities of the preceding three years. Some of you are about to undergo this scrutiny, for others it is yet three years distant, and a few are here who have already answered the inquiry; so I shall not be too communicative and betray our family affairs by disclosing my conclusions, but shall confine myself to the opportunities themselves and their value to you.

Quite within the memory and experience of men not old in the profession it was customary for students of medicine to resort to a year's reading in a doctor's office to acquire a passing familiarity with the appearance of the human bones and a very slight acquaintance with the names and doses of a few drugs. Upon entering college this knowledge was enlarged by actual dissection and a closer study of drugs and their effects, while a very superficial smattering of chemistry and physiology and an attendance upon clinical, medical and surgical lectures filled the remainder of the student's time for the eight or nine months commonly devoted to acquiring a liberal medical education. Of the fundamental studies, anatomy alone was taught by actual laboratory work, and even it was decried by a few men of note as not altogether needed in the equipment of a bold and ready surgeon.

What a different picture is presented today in the elaborate provision made for practical personal study of these formerly condemned branches of medical education! In a course covering four years of eight or nine months each, practically the first two years are spent in laboratory work, which, in itself, does not necessarily belong to medicine alone, but is done equally well in other schools devoted to the study of science in its many forms. Botany, biology, chemistry, anatomy are in no sense bound to such narrow limits as the demands of the medical man; they are branches of science, and are studied here only to bring the student directly to their application to medicine, and to insure the harmony of the knowledge so acquired with that more plainly having a bearing on his professional work. The many weary hours devoted to these so-called fundamental studies grow irksome to the impatient would-be physician; and in his rebellion he is prone to cite his predecessors in practice as living proof of the idleness of such a waste of time and the needlessness of so much abstract information. Indeed, he goes further, and is unfortunately able to say that many reputable schools at this day make no such exacting demands, and while giving the opportunity do not compel the mastery of these studies. Such a superficial view is accepted easily by the men who fail in study, the men who are now getting diplomas by short routes, and by those practical, natural physicians who, instructed in the medicine of twenty years ago, regard advances in their professions as the result of the ceaseless restlessness of the reputation hunter, rather than the outcome of legitimate research. We are forced to stop and ask if this work is useless, if the additional delay in fitting a man for active work is unjustified, and if the old methods were not just as good, inasmuch as they cured sick people to their entire satisfaction. The answer can be put interrogatively by asking if medicine is ever to continue an art, acquired, as in the old barber-surgeon days, by simple imitation, or is it to become at some time a science, whose laws are fixed, and whose students must reach similar conclusions from similar observations. The laws of life may seem inscrutable, they are surely inevitable. Any insight into their workings coupled with the means of detecting the departures from their standard must certainly be of great value to the healer of the sick. I am confident no one would today lay aside the assistance given by a definite knowledge of the laws of chemistry in the study of renal, digestive and vascular diseases, or by an acquaintance with the revelations of the microscope in a daily growing list of diseases.

In the tremendous impetus given to the efforts of the surgeon by the discovery of anesthesia and antisepsis, a knowledge of finer anatomy becomes of the first import; and no longer may the study of grosser anatomy be dismissed with the sneer of over or under familiarity. Nature does her greatest work quietly and in secret places; and an intimate knowledge of the microscopic structures of body is essential to the comprehension of the manner in which disease produces its ravages, and of the nature of the changes themselves. True insight into histology and pathology can not be acquired from the drone of the didactic lecturer, nor from the perusal of text-books; it can only come from the wearying work of the laboratory and the repulsive labor of the dead house.

Time is wanting to cite a bare list of those affections, the positive recognition of which hinges upon knowledge acquired in these studies; and even a longer array is required for the deeds of the physician and surgeon which depend upon information from the same source.

That nobler branch of our profession—preventive medicine—would still be a mere farce and mummery but for the work of the student in those directions which were almost unexplored by our recent predecessors.

Need I say more to evidence the fact that the man possessed of a diploma, acquired without the labor of those early years in the workshop of the schools, has that which may make him a doctor in the eyes of the law, but which can no more make him a physician and a healer of the sick than an ass is transformed by a lion's skin?

Once entered upon one of the most exacting occupations known at the present time; surrounded by obstacles to the quiet pursuit of knowledge; subject to demands upon both time and labor at any moment—scientific investigation becomes difficult, and to most men an impossibility. There is no time now to examine more closely the structure of nerve, muscle or bone in an effort to become familiar with the natural state of those tissues and compare it with the diseased structure presented by the patient seeking relief! Too late to attempt the mastery of the mysteries of the microscope and the attainment of the elusive skill reached only by long and patient toil. In doubt, irresolute, incapable, surrender must be made to the superior skill of those more competent around you, or resort had to the brazen, bluffing assurance of the charlatan—equally impossible to the honest man and destructive to the interests of the patient. There is now rarely any oppoitunity for regaining lost ground; the man is to the world the finished product of the schools; to himself a sham and a fraud.

There is nothing new in this story, nor is it singular to the profession you are entering. The days have gone by for the "natural genius" in mechanics, the born lawyer and the untrained soldier. The world demands more urgently proficiency ever, and looks askance at the claim of the inexperienced to be efficient. There is no doubt that many men wearied of the tiresome monotony of the mimic subcalibre practice of our ships of war in times of peace. There is no doubt that these same men came to know that the trained trigger finger, the sharpened vision, the disciplined subordinate made the difference between an annihilated Spanish fleet at Santiago and an untouched procession of American men of war.

It is idle to multiply instances of so palpable a truth as the value of training and accustomed knowledge. It is more than idle to attempt to prove the consequences of lost opportunity. The man who voluntarily passes by the labor of which he has wearied and beclouds himself in the belief that it doesn't matter, must sooner or later awaken to the fact that his steps must be retraced at immense cost, or that he must ride in the race for life hopelessly handicapped. Some one has said that geniuses in medicine are undesirable, and that the surest basis for good results is hard work and close observation. Reputations are not made in a moment of opportunity by untrained men, and the occasion when it comes can not be met by the unprepared. Isn't it equally true that the ready man seeks and makes the opportunity?

Admiral Dewey was 600 miles from Manila when war was declared, and might possibly have reached the shelter of a home port without a fight. He sailed into the jaws of the Spanish stronghold, made his own opportunity, and demonstrated the usefulness of the months and years of preparation for just such a chance. The man had been made, and needed only a field for the exercise of his abilities. Can any of you number the men in history to whom opportunity came and found them unready? History records deeds, not what might have been, and has rarely stooped to retell the story of the foolish virgins.

It has been said that surely no experience is entirely without value, and while the wisdom of the statement may seem obscure to the student struggling with graphic formulæ, botanical names and the lives and events of bacteria, nevertheless, you can be assured of its utter truthfulness as applied to the drill to which you will be subjected in your approaching studies. No experience is wasted here, and every move on your part is a part and parcel of the final whole; and so carefully is the plan laid for you that any weak point, any neglected opportunity, even if undetected in the close scrutiny of your examinations, will undoubtedly reveal itself when the strain of application comes. Pardon me if I seem to dwell too long on this point. I have been a close observer of medical men, and nothing has more interested me than the effect of early and thorough education upon the after-success of the student. It is granted that men of force have risen in spite of want of advantages in this direction, and have become eminent and respected. It is also conceded that careful education has occasionally resulted in the production of an elaborate prig—full of information, devoid of brain to guide its use. And yet I do not believe that the training of the last man would otherwise than have increased the effectiveness of the first, or that it lessened the usefulness of the second.

In the advanced teaching of the day in medicine I have already spoken of the four-fold increase in the length of time needed to prepare a man for the practice of medicine; and students often deplore their inability to devote the needed time to the study. These men seek such colleges as admit of easy graduation; and, neglecting the fundamental studies, aim at familiarity with the facts immediately needed by the practitioner. Were I the arbiter of such men's future, or doomed to become their patient, I had far rather that they should reverse this arrangement, and ground themselves well in the earlier studies, because, once neglected, these subjects are rarely taken up, while if the later teaching is overlooked, necessity and daily experience will supply the deficiency by slowly erecting a perfect structure on the already secure foundation.

In conclusion, I may be pardoned a direct application of what has been said. This school has not alone aimed at giving thorough clinical instruction in all branches of medicine and surgery, but has laid especial stress upon the fullness, the exactness and care with which the basic subjects are taught. And this has been done in the face of knowledge that it led to lessened classes and a loss of many students, and that no school in the South attempts to maintain an equal standard. The diploma is accorded only finished men; the weak, the incompetent, the idle must seek for their final badge of efficiency in those schools which are willing to forego the labor of preparing a solid foundation. And what of the result? I am entirely willing that the answer should be sought in the records of our graduates in the army, navy and marine hospital service, in the service of the State, and in private practice in competition with the product of the best schools of this country.

